# Draft Genome Sequence of *Bacillus altitudinis* Strain KU-skv2(2), Isolated from a Microbial Mat on an Anthropogenic Pipe from Caldera Uzon (Kamchatka, Russia)

**DOI:** 10.1128/genomeA.01572-17

**Published:** 2018-02-01

**Authors:** Aleksey S. Rozanov, Anton V. Korzhuk, Aleksandra A. Shipova, Alla V. Bryanskaya, Sergey E. Peltek

**Affiliations:** a Federal Research Center Institute of Cytology and Genetics of the Siberian Branch of the RAS, Novosibirsk, Russia

## Abstract

*Bacillus altitudinis* strain KU-skv2(2) was isolated from a microbial mat on an anthropogenic pipe from Caldera Uzon (Kamchatka, Russia, 54°30′0.23″N, 160°0′15.18″E). The sequenced and annotated genome is 3,739,340 bp in size and encodes 3,929 genes.

## GENOME ANNOUNCEMENT

A large number of thermophilic prokaryotes compete for biopolymers in the microbial communities of terrestrial hot springs (e.g., Uzon Caldera, Russia) (1). The species *Bacillus altitudinis* is a microorganism found in air samples (collected at an altitude of 41 km) that was first described in 2006 (2) and later isolated from other environments, including Uzon Caldera. This bacterium produces RNase, which has antitumor and antiviral properties (3). *B. altitudinis* can be used as an efficient microorganism for the enhancement of plant growth and suppression of fungal disease (4).

*B. altitudinis* strain KU-skv2(2) was isolated from a microbial mat on an anthropogenic pipe from Caldera Uzon (Kamchatka, Russia, 54°30′0.23″N, 160°0′15.18″E). We deposited the strain in the collection of biotechnological microorganisms at the Federal Research Center Institute of Cytology and Genetics of the Siberian Branch of the RAS, a source of novel promising objects for biotechnology and bioengineering.

*B. altitudinis* strain KU-skv2(2) culture was cultivated in liquid medium containing 1% tryptone, 0.5% yeast extract, and 1% NaCl. Eight milliliters of cell culture was pelleted by centrifugation and resuspended in 75 µL of H_2_O by intense pipetting. DNA was isolated using the Fermentas DNA purification kit. A NEBNext ultra II DNA library prep kit (New England Biolabs, USA) was used to create libraries for genome sequencing. Genome sequencing was performed on the MiSeq platform (Illumina, USA), using a MiSeq version 3 150-cycle reagent kit (Illumina), at the molecular and cellular biology facility at the Institute of Molecular and Cellular Biology SB RAS (IMCB SB RAS).

*De novo* assembly of the short reads into contigs was performed using SPAdes version 3.10.1 (5). Contigs shorter than 1,000 bp were deleted. A total of 33 contigs yielded a genome sequence of 3,739,340 bp with a GC content of 41.36%. Prediction and automatic annotation of open reading frames was performed using the NCBI Prokaryotic Genome Annotation Pipeline (https://www.ncbi.nlm.nih.gov/genome/annotation_prok). The complete genome sequence carries 3,929 genes, 3,860 protein-coding sequences, rRNAs (1 5S, 5 16S, and 1 23 S), 57 tRNAs, and 5 noncoding RNAs.

### Accession number(s).

The draft genome sequence for *B. altitudinis* strain KU-skv2(2) has been deposited in DDBJ/EMBL/GenBank under the accession no. PEKR00000000. The 33 contigs have been deposited under accession no. PEKR01000001 to PEKR01000033.
